# Estimation of Prediction Intervals for Performance Assessment of Building Using Machine Learning

**DOI:** 10.3390/s24134218

**Published:** 2024-06-28

**Authors:** Khurram Shabbir, Muhammad Umair, Sung-Han Sim, Usman Ali, Mohamed Noureldin

**Affiliations:** 1 Department of Global Smart City, Sungkyunkwan University, Suwon 16419, Republic of Korea or khurramshabbir@alto.fi (K.S.); umairsaif@g.skku.edu (M.U.); ssim@skku.edu (S.-H.S.); 2 Department of Civil Engineering, Aalto University, 02150 Espoo, Finland; 3 Department of Computer Science and Engineering, Sejong University, Seoul 05006, Republic of Korea

**Keywords:** machine learning in SHM, QD-LUBE, uncertainty quantification in SHM, prediction interval

## Abstract

This study utilizes artificial neural networks (ANN) to estimate prediction intervals (PI) for seismic performance assessment of buildings subjected to long-term ground motion. To address the uncertainty quantification in structural health monitoring (SHM), the quality-driven lower upper bound estimation (QD-LUBE) has been opted for global probabilistic assessment of damage at local and global levels, unlike traditional methods. A distribution-free machine learning model has been adopted for enhanced reliability in quantifying uncertainty and ensuring robustness in post-earthquake probabilistic assessments and early warning systems. The distribution-free machine learning model is capable of quantifying uncertainty with high accuracy as compared to previous methods such as the bootstrap method, etc. This research demonstrates the efficacy of the QD-LUBE method in complex seismic risk assessment scenarios, thereby contributing significant enhancement in building resilience and disaster management strategies. This study also validates the findings through fragility curve analysis, offering comprehensive insights into structural damage assessment and mitigation strategies.

## 1. Introduction

The uncertainty quantification (UQ) is an emerging domain, alongside artificial intelligence in natural events where accurate prediction is extremely difficult. To compensate for this uncertainty, a considerable margin over and above the actual requirement of natural disasters is added in structure design which results in a huge cost investment. The nominal design plans and point estimations having insufficient information are unable to address the uncertainty challenges [1]. The main causes of uncertainty include data mismatch, input, and parameter uncertainty. Instead of relying on point forecast value, incorporating an uncertainty margin such as the prediction interval (PI) can help make decisions more credible and reliable [2]. 

One specific example where the challenges of uncertainty are evident is in the design of high-rise buildings situated even in intra-plate regions that face threats from long-period ground motions originating from distant earthquakes. The slow attenuation of long-period waves coupled with potential amplification by soft soil sites renders these structures susceptible to resonance-induced seismic damage. These vulnerabilities have been evidenced during seismic events, such as the 1985 Michoacán earthquake [3], 2011 Tohoku earthquake [4], and 2015 Nepal earthquake [5], whereby high-rise buildings experienced excessive vibrations and severe damage to their non-structural components, notably in Mexico City and Tokyo. This underscores the critical importance of understanding and mitigating the impact of long-period ground motions on high-rise buildings, both structurally and functionally. Given the complex nature of long-period ground motions and the dearth of dependable seismic records, accurate prediction of the structural response of high-rise buildings remains a challenge without real-time monitoring systems supported by sensors and a robust communication infrastructure. The deployment of an early warning system (EWS), as discussed in [6], becomes imperative with access to reliable building data, to ensure decisions are based on the certainty of both the data and the model to mitigate casualties and losses.

The increasing frequency and intensity of earthquakes worldwide highlight the urgent need for advanced methodologies in seismic analysis and SHM. While historically droughts and floods have accounted for significant casualties, the rise in seismic activity, particularly in densely populated urban areas with vertical housing and rapid urbanization, has emerged as a primary concern. Records since the early 1900s indicate a consistent occurrence of major earthquakes, with an average of 16 significant events annually, including one of magnitude 8.0 on the Richter or Mercalli standard scales or greater. The United States Geological Survey (USGS) reports approximately 20,000 earthquakes globally each year with an average of 55 per day [7]. In the past 40–50 years, USGS records show that on average, long-term major earthquakes occurred more than a dozen times every year. Notably, in 2011 alone, 23 major earthquakes of magnitude 7.0 on the Richter or Mercalli standard scales or higher occurred, surpassing the long-term annual average. In other years, the total was well below the annual long-term average of 16 major earthquakes. The lowest-ranking year is 1989 with only 6 major earthquakes followed by 1988 with 7 only. Table 1 shows the top-ranked earthquake countries. These seismic events pose severe threats to structures, particularly those situated near fault lines and seismic zones, resulting in substantial casualties and property losses, amounting to tens of billions of dollars annually.

Post-earthquake probabilistic performance assessment (PPPA) is crucial for promptly and accurately evaluating building safety, particularly in ensuring safe shelter after seismic events. Typically, this assessment is time-consuming and is carried out by licensed engineering experts [9]. Buildings are categorized into safety levels such as inspected, restricted use, and unsafe, based on these assessments [10,11]. However, the scarcity of experts poses challenges, as exemplified by the Tokyo metropolitan government’s 110,375 certified experts tasked with assessing over 1.9 million buildings [11,12]. This shortage becomes more acute during aftershocks or subsequent earthquakes, as demonstrated by the two intense earthquakes that struck the Kyushu area within 28 hours [13]. Thus, the swift and reliable post-earthquake assessment of building structures becomes even more critical in safeguarding human lives. Occupants must be promptly notified of the assessed damage state of the building to facilitate safe evacuation.

## 2. Background and Related Works

In recent research, a novel model for sensor-based EWS and PPPA has been introduced [6], employing the Vanmarcke approximation based on a two-state Markov assumption for extreme value detection. This approach outperforms previous heuristic techniques, demonstrating its superiority. Moreover, advancements in artificial intelligence (AI), particularly machine learning (ML) techniques employing artificial neural networks (ANNs), have garnered significant attention in seismic analysis. These techniques exhibit remarkable accuracy in predicting the transient behavior of buildings, facilitating real-time applications such as EWS and PPPA, as well as informing performance-based design strategies for buildings [14,15]. Notably, AI methodologies have been employed for nonlinear mapping in data modeling, utilizing bootstrapped ANNs for rapid seismic damage evaluation of structural systems [16]. Additionally, AI techniques have been instrumental in stripe-based fragility analysis of multi-span concrete bridges [15] where uniform design-based Gaussian process regression was implemented.

While significant strides have been made in SHM utilizing AI techniques, the crucial aspect of UQ remains largely unexplored in seismic analysis. The oversight of uncertainty can lead to substantial misinterpretations in real-world applications, particularly in scenarios where sudden severe hazards occur. Addressing this gap, researchers have proposed modifications to neural networks (NNs) to account for uncertainty [17,18]. Further advancements include the development of lower upper bound estimation (LUBE) method [19] which integrates delta, Bayesian, bootstrap, and mean-variance estimation (MVE) techniques directly into the NN loss function. While the LUBE technique has gained traction across various domains, such as energy demand and wind speed forecasting, challenges arise during simulation and implementation phases, notably with the risk of converging to a global minimum when all high-quality prediction intervals for deep learning PIs are diminished to zero. To mitigate this issue [17] introduces the quality-driven PI method (QD) and quality-driven ensemble (QD-Ens.), employing gradient descent (GD) standard training methods for NNs, thereby enhancing robustness and reliability in predictive modeling. 

Utilizing QD and QD-Ens. methods, estimating the characteristics of extreme value distribution functions becomes more convenient. Typically, deterministic hazard analysis specifies mean-plus-one-standard-deviation [20]. The QD-LUBE method, known for its high accuracy, rapid convergence, and robustness, is applied to extreme engineering demand parameters (EDPs) like inter-storey drift (IDRs), acceleration (A), and base shear (V) providing prediction intervals (PIs) for observed extreme values. Case studies on a three-storey European laboratory for structural assessment (ELSA) model demonstrate the applicability of this approach. This work signifies a new dimension in assessing building structures in/during post-extreme events, such as earthquakes, enhancing the reliability of probabilistic performance analysis. Correctly estimating value bounds based on analyzed data aids disaster management decision-makers in resource allocation, prioritizing life mitigation, and formulating rehabilitation plans.

## 3. Proposed Method: QD-LUBE-Based Prediction Interval Analysis

The conventional ML techniques like nonlinear mapping in data modeling, utilizing bootstrapped ANNs for rapid seismic damage evaluation, and stripe-based fragility analysis only provide point predictions, which means single output for every single target, and are incapable of monitoring the sampling error, prediction accuracy, and uncertainty of the model. For important decisions and design plans, point estimations cannot provide sufficient information [1]. In seismic analysis, the minimum and maximum values or upper and lower bound values are important for both PPPA and real-time warning systems. Estimating a credible maximum value is crucial as the risk costs the human and capital loss. Furthermore, the uncertainty sources evolved in earthquake prediction must be precisely quantified to provide essential information for decision-makers.

**Figure 1:**
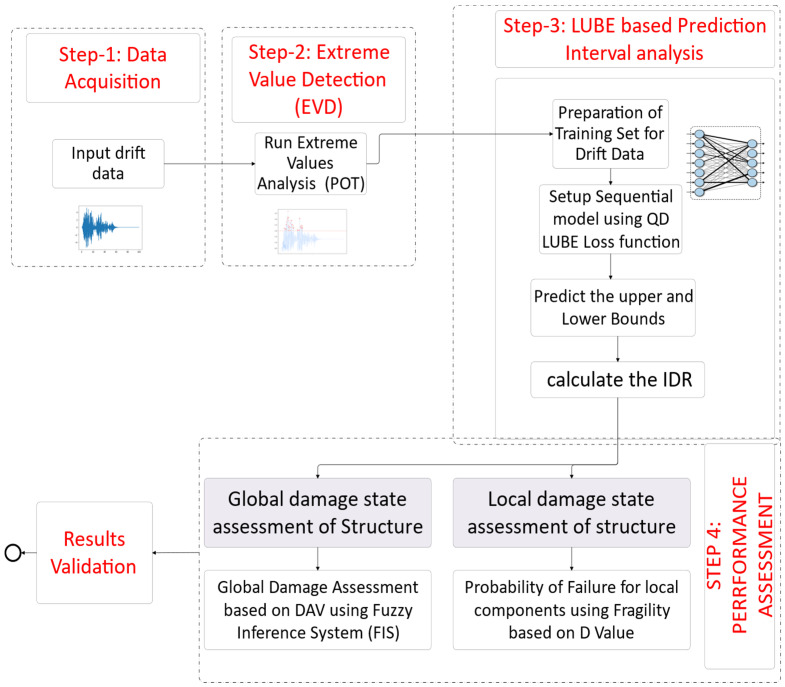
Workflow of the proposed technique.

Hence, in model-based forecasting, specifically the ANN or ML models of natural phenomena, decisions are not solely dependent upon the accurate forecasting the certain variables but also on the uncertainty of data associated with the forecast. The main causes of uncertainty are model and data mismatch, input uncertainty, and parameter uncertainty. Incorporating the uncertainty margins termed as prediction interval (PI) in the determined point forecast value can help to make the decision more credible and reliable [2]. The proposed technique for earthquake damage assessment begins with the data generation and acquisition where response spectra from moderate earthquakes are modeled using the CSI SAP2000 v22 software generating engineering demand parameters (EDPs) such as maximum inter-storey drift (MIDR), acceleration, and base shear. MIDRs are pre-processed for extreme value analysis using the peak-over-threshold method and then prepared for the QD-LUBE method. In the threshold and extreme values detection phase, the generalized pareto distribution (GPD) is employed to identify threshold values, crucial for analyzing extreme values during earthquakes. The QD-LUBE method is then applied to predict the upper and lower bounds of these extreme EDPs, enhancing UQ. The fuzzy inference system (FIS) is subsequently used to assess performance-based damage by associating EDPs with fuzzy membership functions and evaluating them against predefined rules. This process involves fuzzification, rule evaluation, and defuzzification to produce crisp output values. Finally, local and global damage states are assessed using these outputs, with fragility curves and cost estimations based on the upper bounds of EDPs providing a comprehensive framework for earthquake damage assessment and real-time warning systems. An overview of the workflow of the paper has been discussed with minor details. A flow diagram is given in Figure 1.

### 3.1. Data Generation and Acquisition

In the first step, a response spectrum generated from CSI SAP 2000 software with a 475-years’ return period earthquake for a three-storey ELSA model was obtained. We considered the population of moderate earthquakes of the 475-year return period. The model was trained on fifteen moderate-intensity earthquakes to obtain EDPs including IDR, A, and V, collectively called DAV. Further, EDPs data were pre-processed to make them compatible with extreme values analysis using peak-over-threshold (EVA-POT) and subsequently prepared for the QD-LUBE method.

### 3.2. Threshold and Extreme Values Detection

In the case of earthquakes or other natural disasters, extreme values are the main points of interest as they have the worst impact. Being the tail-end values, normal distributions cannot capture them well and the output of the system is normally biased to the overall behavior of the data. For this purpose, GPD was used to select the value of the behavior of data. The approach is used to attain the threshold values for all the earthquakes under analysis. Section 3.3 discusses complete results with certain examples.

### 3.3. QD-LUBE-Based PI Analysis and Uncertainity Framework

In the predictive performance-based earthquake analysis, the concept of UQ is not extensively evolved yet; however, it is gathering fame in other natural hazards like flood and prediction of energy demand. The QD-LUBE method proposed by [17] with fast processing speed, higher accuracy, ease of handling big data, and other competitive benefits over the previous techniques to attain the upper bound of the extreme values has been applied and the model has been trained on the selected earthquake data, i.e., EDPs. The upper bound of EDPs extreme values premeditated in this step have been used in the global and local damage state assessment and global cost estimation.

Pearce et al. [17] proposed the uncertainty framework with QD-LUBE loss function using GD. In the model, if the data generating function *f*(*x*) exist and are combined with additive noise, they produce observable target values y:

*y* = *f*(*x*) + *ϵ* (1)

where ‘*ϵ*’ is termed as irreducible noise or data noise. Some models, for example the delta method, assume ϵ is constant across the input space (homoscedastic), while others allow for it to vary (heteroskedastic). The term “quality-driven” means that the framework incorporates a gradient descent method, designed through qualitative assessment, and includes model uncertainty, which is different than the conventional lower–upper boundary estimation (LUBE) approach. The loss function used in the proposed framework is distribution–free. In other words, it does require any assumption with a specific distribution for the dataset. The loss function (i.e., the objective function) that needs to be minimized to obtain the optimal neural network for a specific dataset can be defined as follows [17]:

(2) $$L=MPIW_{capt}+\lambda \frac{n}{\alpha (1-\alpha )}max[0,\left(1-\alpha \right)-PICP]$$

where $MPIW_{capt}$ is the captured mean prediction interval width, PICP is the prediction interval coverage probability, λ is a Lagrangian multiplier that controls the relative importance of the width compared to the coverage of the prediction interval, n is the number of data points, (1−α) is the desired proportion of coverage, and α is commonly assumed 0.01 or 0.05. The prediction interval (PI) should be bounded by the predicted upper bound, y, and lower bound, $\hat{y}$, such that:

(3) $$Pr({\hat{y}}_{L_{i}}<y_{i}<{\hat{y}}_{U_{i}})(1-\alpha )$$

where $y_{i}$ is the target observation of an input covariate $y_{i}$, and *1* ≤ *i* ≤ *n*. The PI of each point should be calculated such that $MPIW_{capt}$ is minimum, while maintaining PICP <(1−α). To quantify the $MPIW_{capt}$ and PICP mathematically, the following equations can be used:

(4) $${MPIW}_{capt}=\frac{1}{C}\sum_{1}^{n}\left({\hat{y}}_{U_{i}}-{\hat{y}}_{L_{i}}\right)\cdot k_{i}$$

(5) $$PCIP=\frac{c}{n}$$

where c is the total number of data points captured by $PI\left(c=\sum_{i=1}^{n}k_{i}\right),\;{\hat{y}}_{U_{i}},\;{\hat{y}}_{L_{i}}$, and ${\hat{y}}_{U_{i}}$ and ${\hat{y}}_{L_{i}}$ are the upper and lower bounds of the point under consideration, and k_i_ is a binary variable $\left(k_{i}\;\epsilon \;0,\;1\right)$ that represents the occurrence of the data point within the estimated PI, such that:

(6) $$1\;\;\;\;if\;{\hat{y}}_{L_{i}}<\;y_{i}<\;{\hat{y}}_{U_{i}}\;0\;\;\;\;else$$

It is assumed that k can be represented as a Bernoulli random variable (i.e., ki Bernoulli (1 − α)). In addition, $k_{i}$ is assumed to be an independent and identically distributed variable. The former assumption can be used to justify that c can be represented by a binomial distribution (i.e., c binomial (n, (1 − α))). Utilizing the likelihood-based approach, θ, the optimum neural network parameters are optimized to maximize, ${L^{\prime }}_{\theta }=L^{\prime }(\theta |K,\alpha )$, where *K* is the vector of length *n* with each element in the vector is represented by $K_{i}$. Based on the probability, the mass function can be calculated using the central limit theorem and the negative log likelihood.

(7) $${L^{\prime }}_{\theta }=\left(\begin{array}{c} n\\ c \end{array}\right){\left(1-\alpha \right)}^{c}\alpha^{n-c}$$

### 3.4. Fuzzification and Performance-Based Assessment

**Figure 2:**
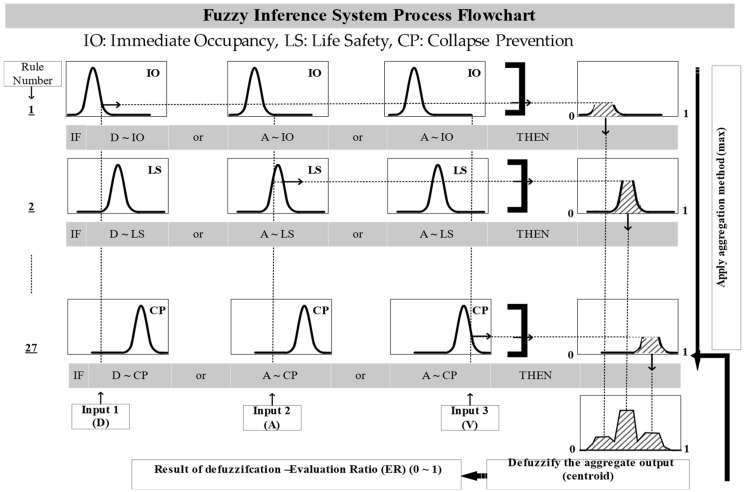
Fuzzy inference system process flowchart.

The fuzzy inference system (FIS) serves a dual purpose in nonlinear mapping within fuzzy logic, addressing inherent fuzziness and vagueness in limit states while relating earthquake damage parameters to multiple limit states simultaneously [21]. Overall, FIS process flowchart is shown in Figure 2. Utilizing the Mamdani procedure, the FIS is established with fuzzification as its initial step, associating each EDP limit state with specific membership functions and determining the degree of association for each EDP value within these functions [22]. Fuzzy operators, employing T-norm (minimum) and S-norm (maximum), are then employed to form fuzzy rules, with the number of rules contingent upon the limit states associated with member functions, which is 27 in our case. The antecedent of each rule is evaluated through fuzzy operators to derive a consequent, a unique number between 0 and 1 [23]. The inference engine assigns implication relations for each rule, allowing for different rule weights to represent their relative importance. This weight assignment is crucial, especially when certain EDPs are of greater significance, necessitating higher weights for corresponding rules. Although in this study, all rules are given equal weight. The maximum composition operator aggregates the output fuzzy numbers from each rule which are then transformed into crisp output numbers through the center of the area (CoA) defuzzification process. This involves dividing the total area of membership function distributions into sub-areas, with the de-fuzzified value (z*) of a discrete fuzzy set calculated based on sample elements and their associated membership functions [22]:

(8) $$z^{*}=\frac{\sum_{1=1}^{n}z_{i}\cdot \mu (z_{i})}{\sum_{1=1}^{n}\mu (z_{i})}$$

where $z_{i}$ is the sample element, *μ*($z_{i}$) is the membership function, and n is the number of elements in the sample [22]. 

The concept of evaluation ratio (ER) and its classification into “recommended”, “moderate”, and “not recommended” classes based on certain threshold values is utilized to assess building damage assessment based on structural characteristics and the earthquake response spectrum. It explains how different ranges of ER correspond to different levels of system performance and suitability for design. These classes are mapped to three different levels of the evaluation ratio as (ER > 0.7), (0.35 > ER > 0.7), and (ER < 0.35), respectively. The ER of each system is labeled “not recommended” if ER < 0.5. The “recommended” class means that system responses are within the recommended limits and vice versa [23,24]. This study aims to compare the fuzzified original, without incorporating the PI uncertainty, on EDPs such as MIDR, A, and V with the enhanced value of EDPs based on PI results.

In this step, local damage state assessment has been performed. The fragility curves of FEMA P-58 PACT software (version 3.1.1) were used as the basis for comparison. Global damage assessment of structures passing the maximum values of DAV through the fuzzy inference system (FIS) is executed, followed by the global cost estimation.

In the local damage state assessment on the maximum inter-storey drift values used, the probability of damage states for post-earthquake performance assessment and real-time warning systems is calculated using the upper bound of extreme values of 15 earthquakes. Global cost estimation is calculated based on D values passed through FIS. Similarly, the global damage state of structures has been calculated using the maximum upper bound values of DAV passing through the FIS. 

## 4. Experimental Evaluation: Case Study Model and Validation

**Figure 3:**
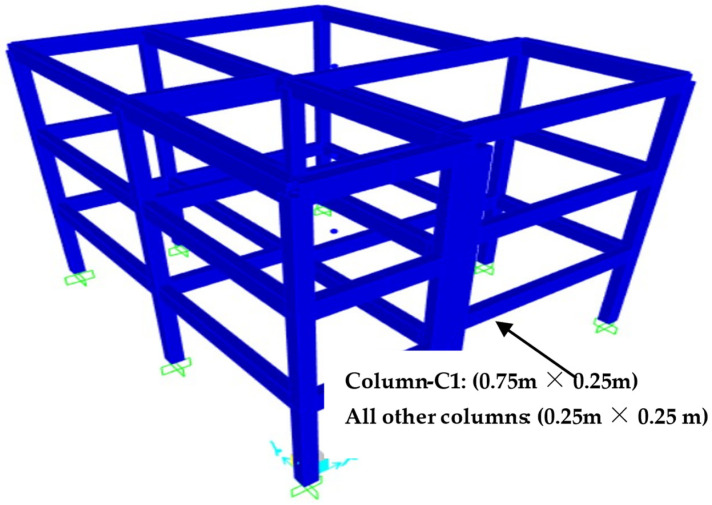
ELSA three-storey model in SAP2000.

All the steps named in the previous section have been elaborated in depth with the ELSA model. The ELSA model is a well-known standard model used as benchmark for most of the structural design software. Components and material details of the ELSA three-storey model are given in Table 2 and Figure 3.

### 4.1. Data Acquisition

**Figure 4:**
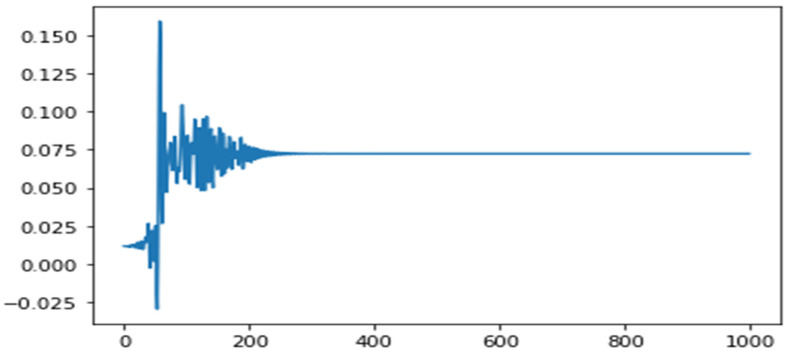
Displacement time series for San Ramon–Eastman Kodak (1980) earthquake.

Non-linear time history analysis (NLTHA) of fifteen moderate-intensity earthquakes with a 475-year return period was used. All these earthquakes have many similar parameters, and huge infrastructure lies on their fault lines. These moderate earthquakes occurred during the years 1956 to 1980 and provide sufficient data. Important parameters of selected earthquakes are given in Table 2. The nonlinear time series data for all the earthquakes have been formulated, pre-processed, and visualized to make it compatible with the model and for other mathematical operations. The nonlinear time series data of MIDR for the first to second floor during the San Ramon–Eastman Kodak (1980) earthquake is plotted in Figure 4. All fifteen earthquakes, as shown in Table 3, were run on this building model in a single degree of freedom (SDOF), i.e., in the Y direction only, to attain the maximum DAV values.

### 4.2. Extreme Values Detection

Hu et al. in [6] used the Poisson’s assumption and compared the results based on the Vanmarcke assumption and Monte Carlo simulation using Kalman Smoother to attain extreme values. However, the extreme values shoot out due to non-flexible, static assumptions only linked with the mean deviation method, leading towards overestimations.

Due to these shortcomings of log-normal distribution and Vanmarcke assumption, the GPD method is used to fit the POT. This allows a continuous range of possible shapes that includes both the exponential and Pareto distributions as special cases. The distribution allows us to “let the data decide” [25] which distribution is appropriate; hence, the highest level of adaptability and accuracy is achieved.

**Figure 5:**
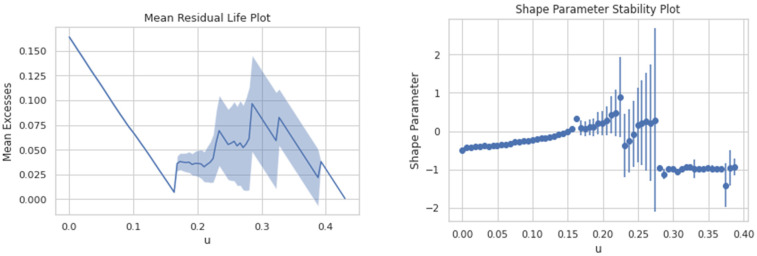
Mean residual life plot and shape parameter stability plot for MIDR third floor to roof for Imperial Valley-07 earthquake.

**Figure 6:**
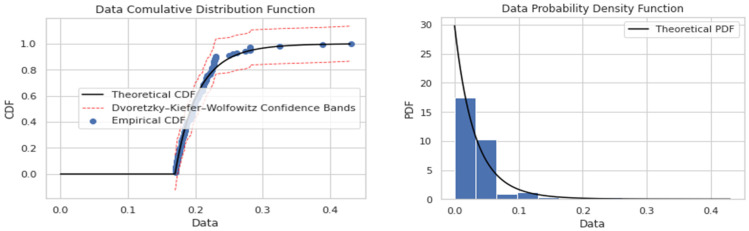
CDF and PDF for mean residual threshold selection.

When fitting the excess with the GPD, the primary problem is the selection of threshold λ. If λ is too large, few excesses and insufficient data lead to excessively large estimator variance. If λ is too small, a large deviation between an excess distribution and GPD leads to a biased estimation. Therefore, a compromise between bias and variance is needed for λ selection. By adopting the straightforward graphic methods including the mean residual life plot and shape and scale parameters stability plots to determine λ based on the average excess function, an optimal threshold value can be calculated separately at each node and for every earthquake. Figure 5 shows the plots of the extreme values data against mean excesses and shape parameters for mean inter-storey drift between the roof and third floor for the Imperial Valley-07 earthquake. Similarly, the are plotted in respect to the cumulative distribution function and probability density function in Figure 6. The threshold selection has been calculated considering the mean value of data for each earthquake. 

Moreover, GPD distribution estimation for IDR values calculated at nodes ‘113’ and ‘112’ for ‘EQ25’(“Imperial Valley-07”, “El Centro Array #7”) is given in Table 4.

### 4.3. LUBE-Based Prediction Interval Analysis

#### 4.3.1. Preparation of Training Sets

**Figure 7:**
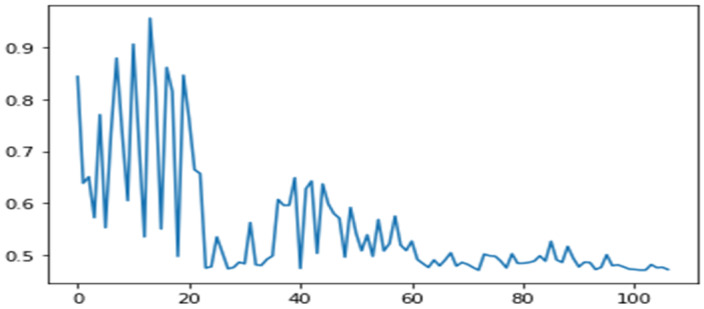
Plot showing IDR extreme values for Livermore–Morgan Terr Park (1980) earthquake at second to third storeys of the ELSA model.

Datasets are the relative joint acceleration, joint displacements, and the sheer force for the 15 earthquakes as explained in Section 2. SAP 2000 produces a nonlinear time series. Pre-processing of data to make them readable to ANN is performed after the extreme values analysis. Absolute values, sorted from the minimum to the maximum value, were used. A sample shape of data is shown in Figure 7.

#### 4.3.2. Setting up the Model

The dataset was further refined and scaled to make it compatible with the model and the loss function of QD-LUBE. The key advantages of the QD-LUBE method are its intuitive objective, low computational demand, robustness to outliers, and lack of distributional assumption. The model used is the Python TensorFlow library Keras sequential model with the input layer and one intermediate layer both having 100 neurons first with RELU activation functions and the output layer having two neurons with the LINEAR activation function. The Adam optimizer was used as a compiler and the confidence level was set at 95%.

#### 4.3.3. Predicting the Upper and Lower Bounds for DAV

**Figure 8:**
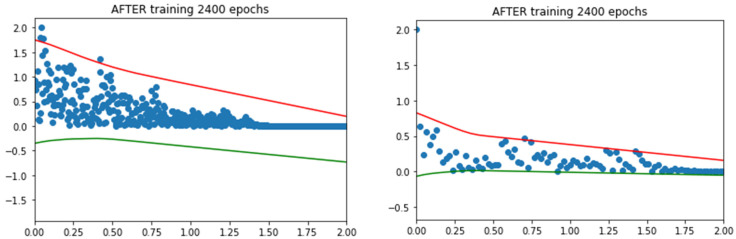
The upper and lower bound of two earthquakes using QD-LUBE method.

Finally, the model was run to make the prediction of the upper and lower bounds. The absolute values were used; hence, the peak values information lies in the upper bound only. The upper bounds of the selected earthquakes are shown in Figure 8. From the graph, we can see the outliers which are abnormal from the distribution; however, PI can determine the upper bound on these outliers using the accumulative behavior of the distribution, and prediction can be calculated for any next value. The same procedure was used to attain the upper bound values of the base sheer and MIDR. Table 5 shows the acceleration values of the maximum value of distribution treated as point prediction, and after fitting the QD-LUBE, an upper bound is calculated for every earthquake. This upper bound adds to the margin of uncertainty while trading itself from the behavior of the input data which is above the threshold value. For comparison, the model was also trained with values more than the median, and it was found that the threshold section method using GPD provides a very good approximation of the extreme events or the tail-end events. The LUBE method further adds to the margin of error as the confidence level is specified in the model (in our case 95%). Therefore, the combination of POT and QD-LUBE provides a very robust hybrid combination of UQ.

The two main parameters used to evaluate the performance of any model based on a statistical model (e.g.,: LUBE method) given in the literature [26] are the normalized mean prediction interval width (NMPIW), which should be minimized, and PICP, which is considered as good as it is near to 1. QD-LUBE is proven to be better than that of Bootstrap or the LUBE method, and this fact was verified by this work. The PICP of the base shear is given in Table 6.

### 4.4. Performance Assessment (Results and Discussion)

In the final step, the building performance assessment was performed for the following aspects:

1. Local damage state assessment using the FEMA P-58 PACT fragility specification manager, using the MIDR values calculated in Section 3.3.
2. Global damage state assessment using the DAV values after passing them through the FIS system of the set limit state member functions.

#### 4.4.1. Local Damage State Assessment

**Figure 9:**
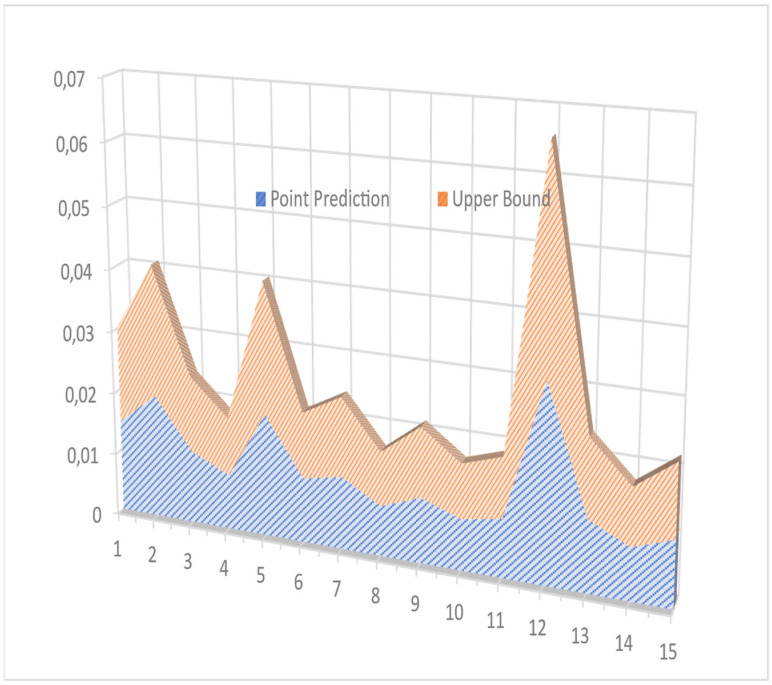
Plot of MIDRs and their upper bound.

The local damage assessment was performed using FEMA P-58 PACT software. Table 7 shows a comparison of the MIDRS point maximum values and the upper bound calculated by the QD-LUBE model. Figure 9 also shows the same graphically. The model provided an uncertainty margin to accommodate for the noise, data, and model uncertainties, closely following the behavior of tail-end data.

**Figure 10:**
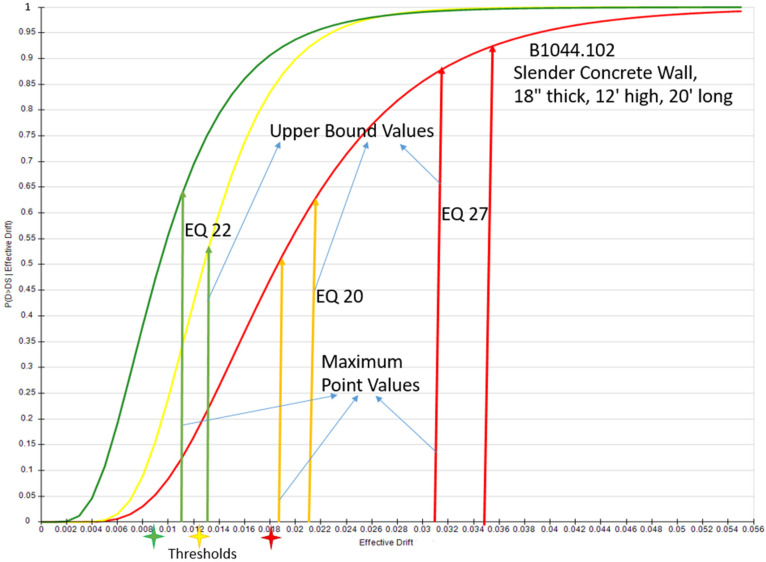
Fragility analysis using the maximum value and upper bound for three earthquakes.

The structural component B1044.102 slender concrete wall, 18” thick, 12’ high, 20’ long, was evaluated on three earthquakes (Imperial Valley-06 at “Chihuahua” station (EQ20), “El Centro Array #12” station (EQ22), and Livermore-01 at “San Ramon–Eastman Kodak” station) as shown in Figure 10. The damage states which were on the borderline were moved to the next damage state after adding the uncertainty margins using the upper bound of the predicted model for each earthquake. Table 7 shows the values of MIDRs simulated by the SAP model and prediction intervals upper bound.

#### 4.4.2. Global Damage Assessment

For the global cost assessment, the value of D_max_ is fuzzified. It is clarified that the highest impact is that of EQ27, followed by EQ17. The ELSA three-storey model has very minor to no damage in the case of other earthquakes. Results of fuzzification are given in Table 8. 

For UQ, the global damage assessment of DAV based on PI has been fuzzified. The evaluation ratio (ER) of EQ-17 and EQ-27 shows the highest damage. Structures’ evaluation for these earthquakes resulted in “not recommended”. Hence, structural parameters need to be modified to attain performance assessment results in the acceptable range. Table 9 provides normalized and un-normalized output values and evaluation ratios after fuzzification. 

## 5. Conclusions

The distribution-free ensemble approach, the QD-LUBE method, has been used for uncertainty quantification, in assessing the critical parameters of structural models like inter-storey drift, peak ground acceleration, and base shear, which has been proven as powerful ML tool. In this study, the QD-LUBE was applied to the ELSA model, focusing on a three-storey building subjected to 16 well-known earthquakes within a single degree of freedom framework.

Key aspects of the methodology included leveraging the Vanmarcke’s assumption for extreme value detection, which allowed us to extract peak-over-threshold determinations. The upper bounds of prediction intervals for certain parameters were accessed by training the model and testing on given datasets. The process yielded robust results and demonstrated superior performance compared to the bootstrap method in terms of accuracy and reliability.

Furthermore, the findings were validated through fragility curve analysis, specifically evaluating the impact of three earthquakes on the effective drift and transitioning between damage states. To assess the overall structural damage, a fuzzy inference system was integrated, providing a comprehensive evaluation of the global damage state.

The research not only contributes to advancing the field of uncertainty quantification in structural engineering, but also showcases the efficacy of the QD-LUBE method in handling complex scenarios and providing actionable insights for seismic risk assessment and mitigation strategies.

## 6. Future Works

The LUBE method can be used to enhance the reliability of a real-time warning system by predicting the upper bound of point prediction. In the event of an earthquake, the system will take the data from sensors and ICT infrastructures. The data samples from multiple buildings can help to make the decision for all the buildings of the same kind and the model trains itself with the extreme values behavior to provide certain values. Moreover, novel ML models are considered to improve the seismic performance assessment of buildings by incorporating additional details and methodologies [27,28]. Determining a threshold in peak-over-threshold modeling using mean residual life and threshold stability plots involves significant subjectivity. The identification of linear portions in these plots is challenging due to the vague definition of linearity, leading to potential errors in selecting constant scale and shape parameter estimates. An objective method like a segmentation approach is needed to accurately determine the constant portion of these parameters [29]. 

## Figures and Tables

**Table 1:** Ranking of most earthquake-prone countries [8].

Rank	1	2	3	4	5	6	7	8	9
**Country**	China	Indonesia	Iran	Turkey	Japan	Peru	USA	Italy	Afghanistan, India, Greece, and Mexico
**No. of earthquakes, from 1900 to 2016**	157	113	106	77	61	44	41	33	32

**Table 2:** Steel (**a**) and concrete (**b**) properties of ELSA model.

a. Steel properties		
**Material**	$F_{y}\;(KN/m^{2})$	$F_{u}\;(KN/m^{2})$
A992Fy50	458,999.89	517,106.84
b. Concrete properties		
**Material**	$F_{c}\;(KN/m^{2})$	$eF_{c}\;(KN/m^{2})$
4000Psi	27,579.03	27,579.03
C25/30	25,000	25,000

**Table 3:** List of the selected earthquakes.

EQ NO.	Earthquake Name	Year	Station Name	Magnitude
16	“El Alamo”	1956	“El Centro Array #9”	6.8
17	“Hollister-02”	1961	“Hollister City Hall”	5.5
18	“Borrego Mtn”	1968	“El Centro Array #9”	6.63
19	“Borrego Mtn”	1968	“San Onofre-So Cal Edison”	6.63
20	“Imperial Valley-06”	1979	“Chihuahua”	6.53
21	“Imperial Valley-06”	1979	“Delta”	6.53
22	“Imperial Valley-06”	1979	“El Centro Array #12”	6.53
23	“Imperial Valley-06”	1979	“Westmorland Fire Sta”	6.53
24	“Imperial Valley-07”	1979	“El Centro Array #4”	5.01
25	“Imperial Valley-07”	1979	“El Centro Array #7”	5.01
26	“Imperial Valley-07”	1979	“El Centro Array #8”	5.01
27	“Livermore-01”	1980	“San Ramon-Eastman Kodak”	5.8
28	“Livermore-01”	1980	“Tracy-Sewage Treatm Plant”	5.8
29	“Livermore-02”	1980	“Livermore-Fagundas Ranch”	5.42
30	“Livermore-02”	1980	“Livermore-Morgan Terr Park”	5.42

Fault Mechanism: Strike-slip.

**Table 4:** GPD distribution estimation for IDR values calculated at nodes ‘113’ and ‘112’ for ‘EQ25’(“Imperial Valley-07”, “El Centro Array #7”).

Estimator: MLE Deviance: −471.854 AIC: −467.854		Threshold Call: 0.17 Number above: 102 Proportion above: 0.1021		Optimization Information Convergence: Successful Function Evaluations: 38 Gradient Evaluations: 6	
Estimates		Standard Errors		Asymptotic Variance Covariance	
Scale	Shape	Scale	Shape	Scale	Shape
0.03352	0.08286	0.004622	0.096623	2.136 × 10^−5^ −2.831 × 10^−4^	−2.831 × 10^−4^ 9.336 × 10^−3^

**Table 5:** Maximum point prediction and upper bound of the acceleration values.

EQ	Roof Top (PI)	Max. Point Prediction	Second Floor (PI)	Max. Point Prediction	First Floor (PI)	Max. Point Prediction
16	2.9675	3.4174	5.9089	5.2019	6.215	5.345
17	2.8001	3.4675	6.8572	5.5788	9.3251	7.8572
18	2.3061	2.6807	4.8099	4.2846	5.2393	4.5784
19	3.0411	3.4264	6.2884	5.5184	7.3024	6.4267
20	3.4903	4.138	6.655	5.5865	8.2102	6.9327
21	3.1628	3.6077	5.7879	5.247	7.1848	6.2229
22	3.1478	3.7681	4.6042	3.9615	7.4352	6.5044
23	2.2957	2.7041	4.1963	3.6141	5.7631	4.9219
24	9.8914	10.7104	11.5897	10.7372	11.7686	10.6755
25	4.1296	4.8969	8.9263	8.5162	7.7993	6.9579
26	5.2757	6.2128	9.4837	8.8617	10.3545	9.3089
27	3.0524	3.5914	6.1852	5.2417	8.3252	7.2863
28	1.8029	2.1028	3.7836	3.2533	4.7728	3.9677
29	7.4119	8.4068	8.3186	7.4626	10.6357	9.3142
30	4.337	5.0773	10.1222	8.6577	8.2177	6.8408

**Table 6:** PCIP of the base shear of all the selected earthquakes.

EQ No.	16	17	18	19	20	21	22	23	24	25	26	27	28	29	30
**PICP**	0.9637	0.9899	0.9779	0.9899	0.9822	0.9584	0.9498	0.9617	0.9897	0.9962	0.9632	0.9658	0.9758	0.9604	0.9774

**Table 7:** MIDR values and upper bound for 15 earthquakes.

Earthquakes	16	17	18	19	20	21	22	23	24	25	26	27	28	29	30
**MIDR** **point value**	0.0147	0.0198	0.0116	0.0087	0.0195	0.0103	0.0115	0.0079	0.0104	0.0081	0.0092	0.0312	0.0113	0.0082	0.0105
**MIDR LUBE** **Upper Bound**	0.0154	0.0211	0.0122	0.009	0.021	0.011	0.013	0.009	0.011	0.009	0.010	0.035	0.012	0.009	0.012

**Table 8:** ER of engineering demand parameters (DAV) values’ fuzzification without PI.

EQ	16	17	18	19	20	21	22	23	24	25	26	27	28	29	30
ER	50	47.7049	50	50	50	50	50	50	50	50	50	37.7852	50	50	50

**Table 9:** ER for engineering demand parameters (DAV) values fuzzification based on PI.

	OUTPUT						FIS
Earthquakes	PI Non-Normalized			PI Normalized			
	D	A	V	D	A	V	
	MIDR	g	kips	_	_	_	ER
16	0.015462	0.633537	150.2882186	0.293871	0.510105556	1	37.7852
17	0.021186	0.950571	182.280015	0.402999	0.843064627	1	11.6554
18	0.012242	0.534077	151.4524366	0.232481	0.405649697	1	37.7852
19	0.009	0.744	138.406	0.176468	0.626519721	1	37.7852
20	0.021	0.837	167.104	0.399739	0.723706385	1	37.7852
21	0.011	0.732	141.637	0.19927	0.613929776	1	37.7852
22	0.013	0.758	142.793	0.23986	0.640736937	1	37.7852
23	0.009	0.587	119.586	0.162684	0.461726338	1	37.7852
24	0.011	1.200	133.100	0.209661	1.104659268	1	11.6539
25	0.009	0.910	120.495	0.164229	0.800370155	1	37.0901
26	0.010	1.056	110.478	0.180205	0.953269466	1	11.6539
27	0.035	0.849	218.384	0.661673	0.736017981	1	37.7852
28	0.012	0.487	143.498	0.234483	0.355707442	1	37.7852
29	0.009	1.084	118.425	0.17216	0.983373993	1	11.6539
30	0.012	1.032	127.581	0.219593	0.928400043	1	11.6539

## Data Availability

The data presented in this study are available on request from the corresponding author due to privacy.
